# Cortical Resonance Frequencies Emerge from Network Size and Connectivity

**DOI:** 10.1371/journal.pcbi.1004740

**Published:** 2016-02-25

**Authors:** Caroline A. Lea-Carnall, Marcelo A. Montemurro, Nelson J. Trujillo-Barreto, Laura M. Parkes, Wael El-Deredy

**Affiliations:** 1 Faculty of Medical and Human Sciences, University of Manchester, Manchester, United Kingdom; 2 Faculty of Life Sciences, University of Manchester, Manchester, United Kingdom; 3 School of Biomedical Engineering, University of Valparaiso, Valparaiso, Chile; Hamburg University, GERMANY

## Abstract

Neural oscillations occur within a wide frequency range with different brain regions exhibiting resonance-like characteristics at specific points in the spectrum. At the microscopic scale, single neurons possess intrinsic oscillatory properties, such that is not yet known whether cortical resonance is consequential to neural oscillations or an emergent property of the networks that interconnect them. Using a network model of loosely-coupled Wilson-Cowan oscillators to simulate a patch of cortical sheet, we demonstrate that the size of the activated network is inversely related to its resonance frequency. Further analysis of the parameter space indicated that the number of excitatory and inhibitory connections, as well as the average transmission delay between units, determined the resonance frequency. The model predicted that if an activated network within the visual cortex increased in size, the resonance frequency of the network would decrease. We tested this prediction experimentally using the steady-state visual evoked potential where we stimulated the visual cortex with different size stimuli at a range of driving frequencies. We demonstrate that the frequency corresponding to peak steady-state response inversely correlated with the size of the network. We conclude that although individual neurons possess resonance properties, oscillatory activity at the macroscopic level is strongly influenced by network interactions, and that the steady-state response can be used to investigate functional networks.

## Author Summary

When entrained using repetitive stimulation, sensory cortices appear to respond maximally, or resonate, at different driving frequencies: 10Hz in visual cortex; 20Hz and 40Hz in somatosensory and auditory cortices, respectively. The resonance frequencies are inversely correlated to the cortical volume of the respective regions, but it is unclear what drives this relationship. Here we used both computational and empirical data to demonstrate that resonance frequencies are emergent properties of the connectivity parameters of the underlying networks. The experimental paradigm stimulated large and small areas of visual cortex with different size objects made of flickering dots, and varied the driving frequency. Larger cortical areas exhibited maximum response at lower frequency than smaller areas, suggesting the inverse relationship between cortical size and resonance frequency holds, even within the same sensory modality. Computationally, we simulated cortical patches of different sizes and varied their connectivity parameters. We demonstrate that the size of the activated network is inversely related to its resonance frequency and that this change is due to the increased transmission delay and greater node degree within the larger network. The results are important for understanding the functional significance of oscillatory processes, and as a tool for probing changes in functional connectivity.

## Introduction

Although oscillations are observed in the human electroencephalogram (EEG) within a wide range of frequencies (1–100 Hz), each primary sensory cortex responds maximally to a specific frequency when driven by external repetitive stimuli using the steady-state evoked potential (SSEP). The SSEP is an experimental method of ‘tagging’ cognitive processing using a repetitive stimulus where the frequency response of the tag is augmented in the EEG spectrum and varies by task condition [1,2]. The driving frequency evoking the largest steady-state response, also known as the ‘resonance frequency’ [3,4], depends on which of the sensory cortices is being stimulated. In the visual cortex, the SSEP is greatest when the driving frequency is in the range of 10-18Hz, whereas in the somatosensory and auditory cortices the maximum response is between 20-26Hz and 38-42Hz, respectively [3,5–10]. This could be a mechanism by which each of these systems is able to extract sensory information optimally from its environment [4]. There is an inverse relationship between the size of each primary sensory area and its corresponding resonance frequency range. Anatomical studies have quantified the volume of the primary visual cortex in humans (Brodmann Area 17) to be approximately 23 *cm*^3^; that of primary somatosensory cortex (Brodmann Areas 1, 3a and 3b) to approximately 13 *cm*^3^; and of the auditory cortex (Brodmann Area 41) to approximately 3 *cm*^3^ [11–13].

It is not clear whether the observed inverse relationship between the size of a sensory region and its resonance frequency is due to differing physiological characteristics of the underlying neural components within each region, or due to emergent properties of the neuronal network connecting them. In physics, Mersenne’s Law is an inverse relationship between the fundamental frequency of a vibrating string and it length [14]. It is plausible that the frequency of oscillations within a cortical network is proportional to the length of the axons that connect them. Computational results from the study of synchronisation in networks of phase oscillators support the hypothesis that topological aspects of the network affect the oscillatory behaviour of individual units. Nordenfelt *et al* [15] found that the length of the coupling delays and degree of connectivity were negatively correlated with the oscillatory frequency of units within the network. These results raise the possibility that a similar relationship exists between the resonance frequency of a neural network and its connectivity parameters. We explored these theoretical results in an oscillator model designed to better describe neural activity and then extended the work to include experimental validation of these findings. Comparing resonance properties of functionally different brain regions becomes problematic due to the many structural and physiological differences that exist. Therefore, in this paper we chose to investigate network resonance within the same brain area and restrict our analysis to homogenous networks.

Initially, we implemented a model of loosely-coupled Wilson-Cowan (WC) oscillators intended to simulate a small patch of human primary visual cortex [16–18] to confirm the hypothesis that network size is inversely correlated with resonance frequency. In the human brain, anatomical architecture is fixed. Functional neural networks arise transiently according to the task being performed [19]. They are themselves sub-networks within the global network of the brain or brain-region within which they reside and are constrained by the physical connections already in existence. Therefore, it is impossible to completely isolate a cortical network from all external activity. Instead, neurons fire coherently maximising the efficiency of communication between synchronous units [20]. Therefore, we activated sub-networks of increasing size using repetitive stimuli allowing us to measure resonance. The network was designed to incorporate lateral inhibition of units surrounding the activated area [21,22]. The modelling work indicated an inverse relationship between network size and resonance frequency. Since network size also affects the average length of the transmission delays and degree of connectivity in a homogenous network, we conducted further simulation studies to describe the specific effect of each of these parameters on the resonance frequency of the network. The term ‘delay’ in this manuscript is defined as the interval of time that it takes a signal to propagate along the axon from one unit to another. For the purposes of our study, we assume that the conduction velocity is fixed and therefore the delay is proportional to the distance between two connected units.

**Fig 1:**
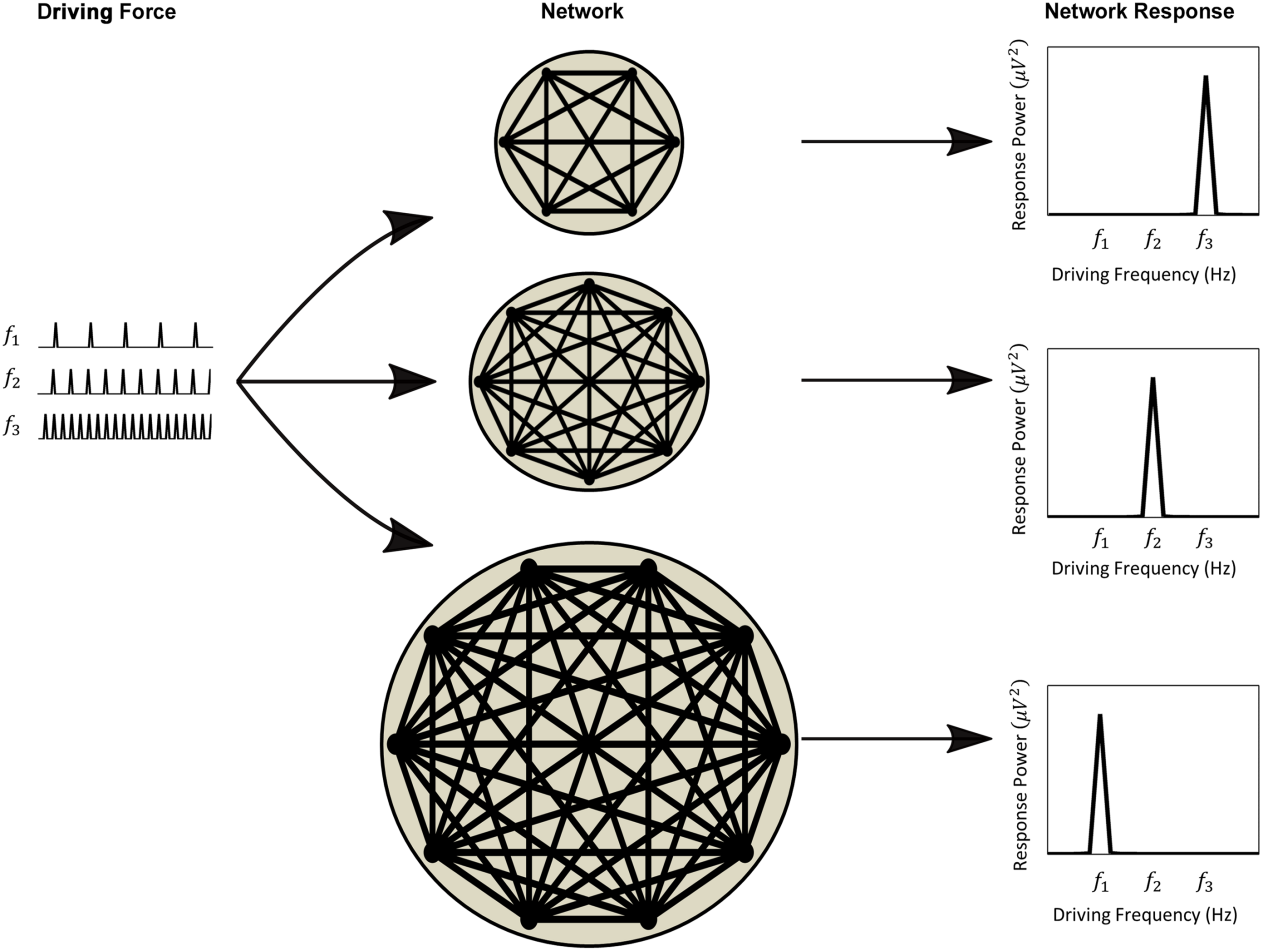
Size matters: A schematic of the hypothesised effect of network size on resonance frequency. Networks of loosely coupled oscillators of different size exhibit different resonance characteristics that manifest as peaks in their spectral response. One method to probe the resonance properties is to drive the networks with repetitive, periodic stimuli over a range of frequencies (*f1 < f2 < f3*). For each network, the driving frequency corresponding to the maximal response power is deemed to be closest to the resonance frequency of that network. For example, f3 corresponds to the resonance of the top network, while f1 is the resonance if the bottom network. Here we test the hypothesis that as network size increases, the resonance frequency monotonically decreases.

Finally, we tested the model predictions experimentally using the visual SSEP (see Fig 1 for a schematic diagram of the experimental paradigm). Stimuli of different sizes, thus activating different sized patches of the primary visual cortex, flickered at different driving frequencies. The stimuli design and their presentation ensured that there was no change in the physiological properties of the constituent neurons of each of the activated networks. This allowed us to examine intra-subject differences in network resonance thereby controlling for biophysical factors such as individual myelination. The results confirmed the prediction of the model that the larger the size of the activated visual cortical network, the lower the resonance frequency. We suggest that resonance can be utilised as a marker for change in the underlying neural networks within the brain following disease, natural development or trauma.

## Results

### Computational Results

We studied the change in resonance frequency in networks of different sizes using a homogenous network model of loosely-coupled identical Wilson-Cowan oscillators (see Materials and Methods). We first quantified the extent to which the resonance of a sub-network within our model was affected by its size. We also considered the effects of increasing transmission delay and node degree on the resonance frequency. The model prediction was that the relationship between size and resonance frequency would be preserved in cortical networks.

### Resonance Characteristics of Sub-networks within the Model

**Fig 2:**
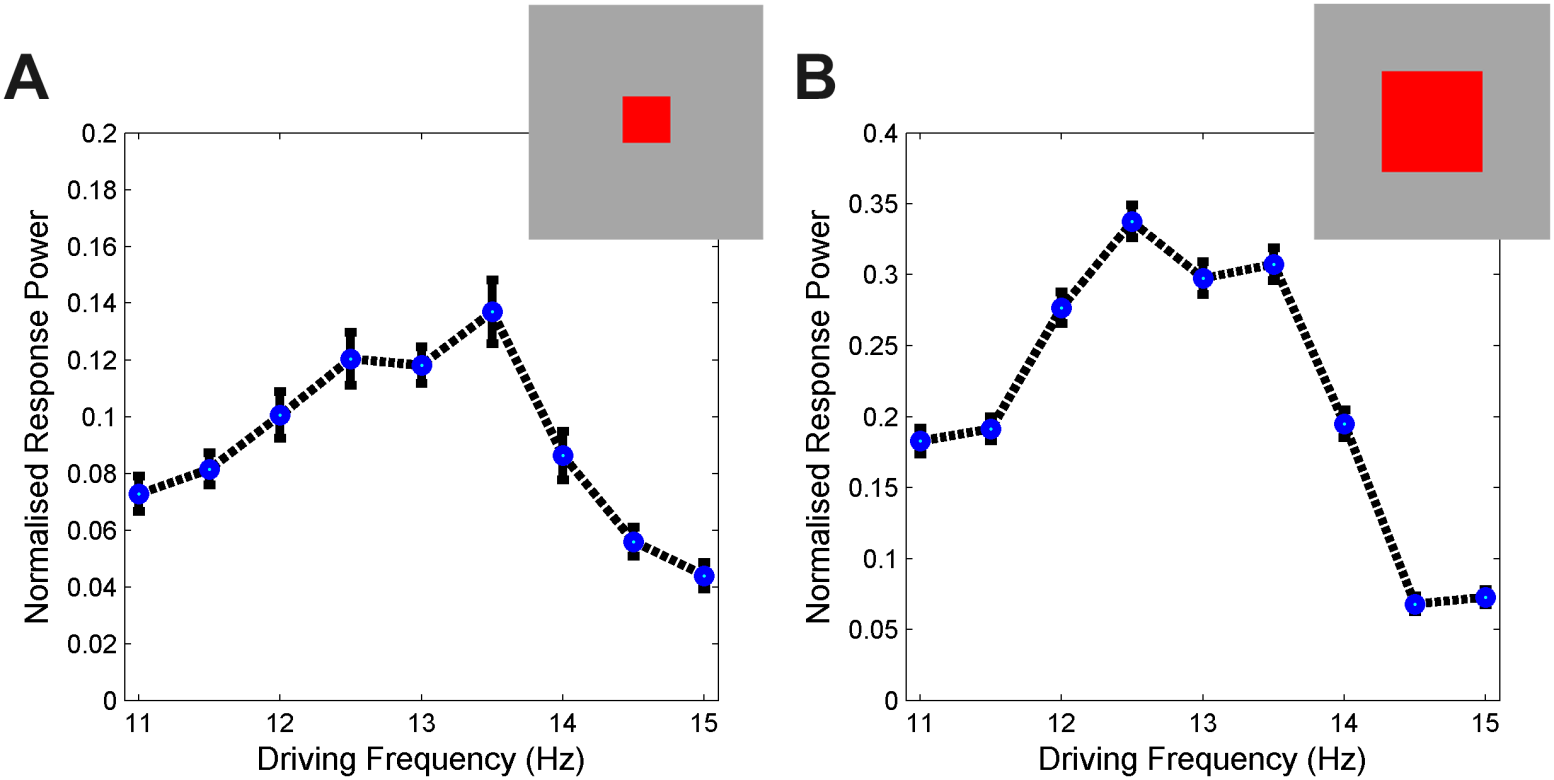
Resonance frequency change with sub-network size. Two biophysically plausible sub-networks (red patches, A 10x10; B 30x30) within a larger Wilson-Cowan network of loosely-coupled oscillators (grey patch, 50x50 units) were driven by external inputs with frequencies varying between 11 Hz and 15 Hz. Mean response power over 100 trials at each frequency calculated using the bootstrap method (see Materials and Methods), normalised with respect to the total power per trial, is given in blue with standard deviation as black bars. The frequency of maximal response for the small sub-network is 13.5 Hz and for the large network it is 12.5 Hz. Repeated measures ANOVA showed only a significant frequency and network size interaction (F = 479.03, p<0.0001) suggesting that the frequency of the maximum steady-state response depended on network size. See S1 Fig for a demonstration of the response properties of a single unit to a range of external frequencies and the relationship between the frequency of maximal response power to the natural peak-frequency of that unit as a response to white-noise input.

Two different sized network patches were stimulated with driving frequencies between 11 and 15 Hz. In Fig 2, the mean response power for each condition over 100 trials using the bootstrap method (see Materials and Methods), normalised with respect to the total power per trial, is given in blue with the standard deviation shown as black bars. We observed a shift in the peak response from 13.5 Hz in the small network to 12.5 Hz in the larger network. A two-way repeated measures analysis of variance (ANOVA) with factors driving frequency and network size was performed on the two peak frequencies 12.5 Hz and 13.5 Hz. There was no main effect of driving frequency (F = 0.07, p = 0.83) or size (F = 70.25, p = 0.08) on response power, but a significant interaction effect was found (F = 479.03, p<0.0001). There is a trend towards a main effect of size and this is due to the greater signal-to-noise ratio achieved in the larger network.

### Impact of Network Properties on the Resonance Frequency

**Fig 3:**
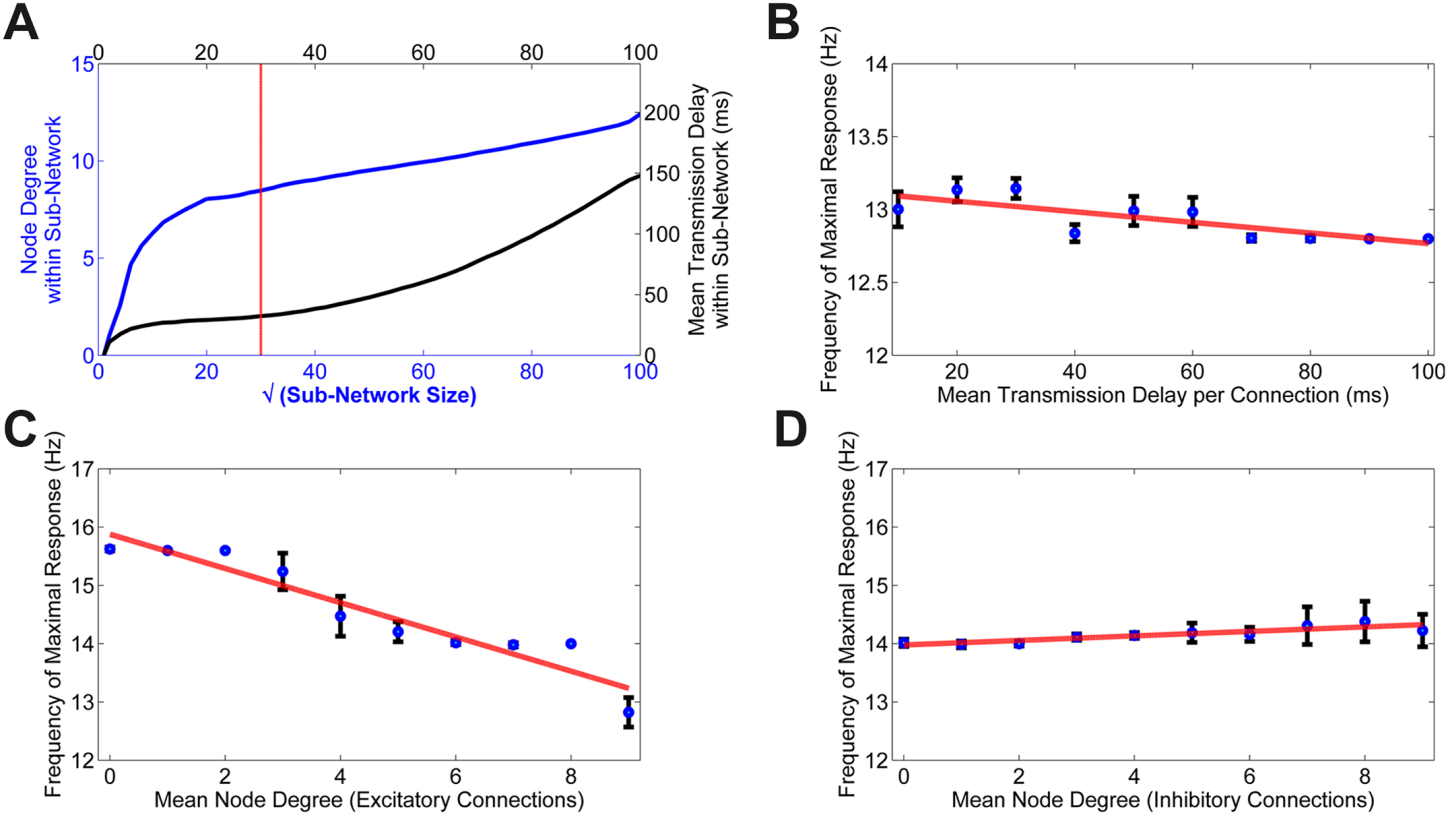
Resonance frequency depends on connectivity properties. **A** Increasing the size of sub-networks (from 2x2 to 100x100) increased the two main connectivity properties: average node-degree and average transmission delay. Node degree is measured as the number of active connections (either excitatory or inhibitory) between all units contained within the specified sub-network. **B** Resonance Frequency as a function of varying transmission delay between units (*R*^2^ = 0.62, *p* < 0.01). For the purposes of our study, we assume that the conduction velocity is fixed and therefore the transmission delay is proportional to the distance between two connected units on a Euclidean plane. **C-D** The effect of node degree on resonance. The mean node degree is defined as being the average number of connections made from one unit to another. We test the effect of increasing local node degree for each of the two types of local connections (excitatory and inhibitory). To achieve this, the local inhibitory (excitatory) connections were fixed to a value of 5 and the node degree for the opposite connection type was increased from 0 to 9, given in **C** (*R*^2^ = 0.91, *p* < 0.0001), **D** (*R*^2^ = 0.80, *p* < 0.001).

Certain properties of our network were observed to be dependent on size; specifically the mean node degree and transmission delay (see Fig 3A). When the network is small, both of these parameters grow steeply with increasing size, while for larger networks both curves grow more slowly. This points to the presence of a microscopic regime, within which the size of the network is comparable to the width of the Gaussian associated with the extent of short-range connections, and a macroscopic regime in which the sub-network size is significantly larger than the range of local connections. Given this relationship between the size of the network and both node degree and transmission delay, we considered the effect of each of these parameters on the resonance of the network.

### Fixed Node Degree

For simplicity of simulation we first fixed the mean node degree, along with all other parameters within the model (see Table 1), while transmission delay was varied in proportion to distance from 10 to 100 time steps. A longer delay implies a larger network in terms of distance between the units. We found that longer transmission times were associated with a lower resonance frequency (Fig 3B) (*R*^2^ = 0.62, *p* < 0.01).

**Table 1:** Parameter values.

Figure Number	Network Size	Noise Level	Inter-Unit Excitatory Connection Strength	Inter-Unit Inhibitory Connection Strength	Transmission Delay (per unit distance)	Mean Node Degree (Excitatory)	Mean Node Degree (Inhibitory)	Long-Range Connections (Excitatory)	Entraining Frequency
2	50x50	0.05	0.15	0.10	10	5	5	~25% of local connections	varied between 11 Hz and 15 Hz
3A	100x100	N/A	N/A	N/A	10	5	5	~25% of local connections	None
3B	30x30	0.05	0.15	0.10	varied between 10 and 100	5	5	~25% of local connections (fixed at mid-value)	varied between 12 Hz and 14 Hz
3C	30x30	0.07	0.15	0.10	0	Varied between 0 and 9	5	~25% of local connections (fixed at mid-value)	varied between 12 Hz and 17 Hz
3D	30x30	0.07	0.15	0.10	0	5	Varied between 0 and 9	~25% of local connections (fixed at mid-value)	varied between 12 Hz and 17 Hz

### Fixed Delay

Next, the effects of increased local node degree, the number of local connections per node or unit, were tested separately. In this simulation we assumed instantaneous transmission of information to ensure that there was no increases in mean delay as the number of connections grew. The mean node degree is defined as the average number of connections per unit. In the following simulations, we define excitatory (inhibitory) node degree to describe the average number of forward local excitatory (inhibitory) connections for each unit. We distinguished between the effects of the two types of local connections: excitatory and inhibitory by fixing one at a value of 5 and varying the other between 0 and 9. Long-range connections remained fixed. When the number of local excitatory connections per unit was fixed, the effect of increasing the inhibitory connections had a positive correlation with network resonance, Fig 3C (*R*^2^ = 0.91, *p* < 0.0001). Conversely, when the number of local inhibitory connections per unit was fixed the degree of excitatory connections had a strong negative relationship with frequency, Fig 3D(*R*^2^ = 0.80, *p* < 0.001).

### Experimental Results

**Fig 4:**
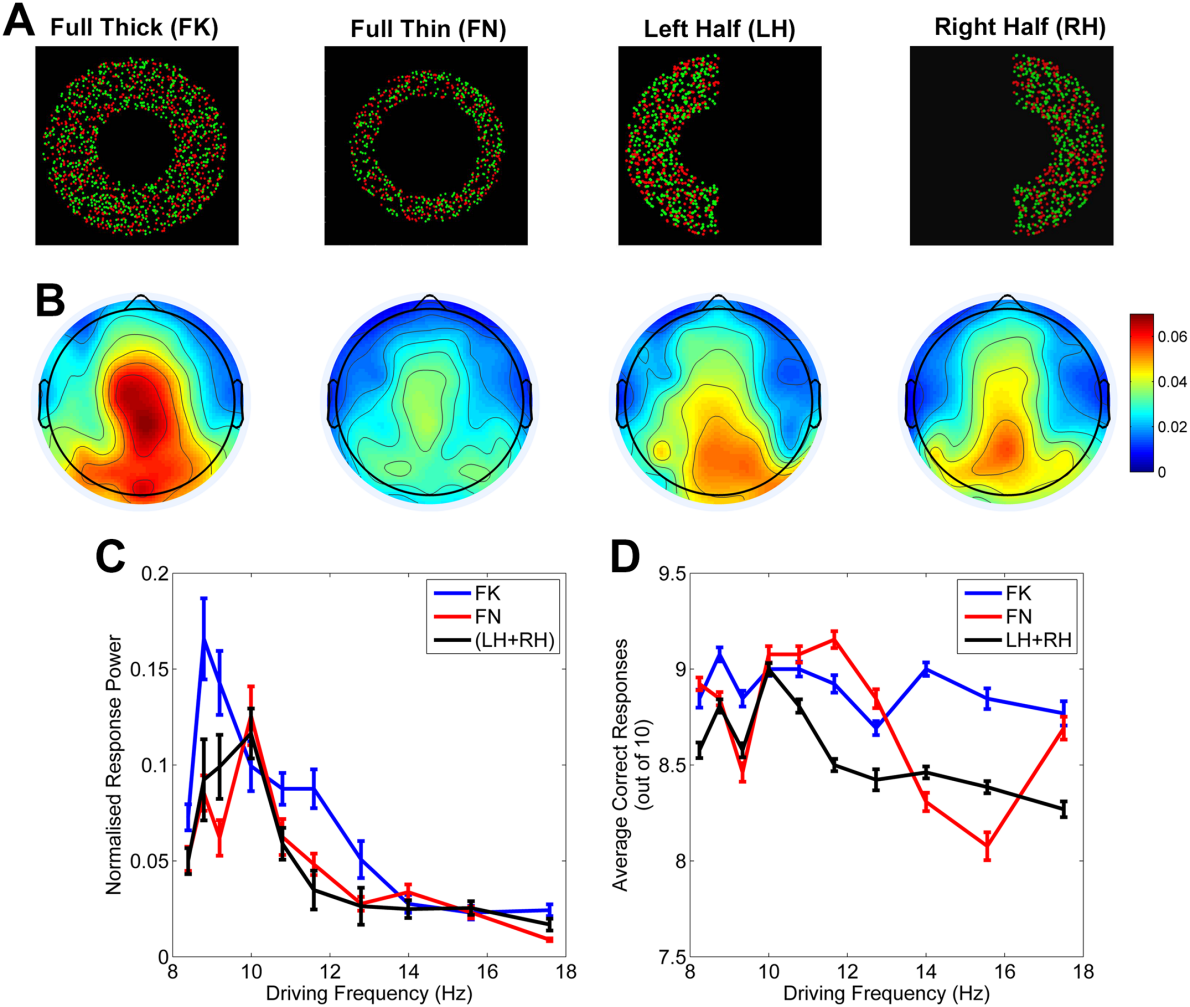
Experimental results. **A** The 4 visual stimuli. Each image was presented flashing at the 10 driving frequencies 10 times each. The images consisted of randomly placed green and red dots, one of which was dominant. Participants were asked to fixate on the centre of the screen and discriminate which was the dominant colour from the flashing image in their periphery. The Full Thin stimulus (FN) was equal to exactly half the total area of the Full Thick stimulus (FK), as were the Left Half (LH) and Right Half (RH) stimuli. **B** Grand average normalised scalp topographies are given for the four conditions at a single stimulus frequency (9.3 Hz). The power at the stimulus frequency normalised by the total power at each electrode is given. **C** Normalised mean response power, and standard error, at the driving frequencies per stimulus type. The maximum response for the FK case is at 8.8 Hz and at 10.0 Hz for FN and (LH+RH). Statistically significant response differences for FN condition as compared to FK were for 8.8 Hz, 9.3 Hz and 17.5 Hz. For the (LH+RH) as compared to FK statistically significant differences in response power were found for 8.8 Hz, 9.3 Hz and 11.7 Hz. **D** Mean percentage of correct responses for each stimulus type at each frequency with standard errors given as bars. Individual scores ranged between 40% and 100% across all conditions.

We created visual stimuli of different sizes that were designed to activate specific target patches within the visual cortex in order to mirror the experimental work. This was possible due to the precise nature of retinotopic mapping of the visual field onto the cortex [23]. Four image types were presented, a Full Thick annulus (FK), a Full Thin annulus (FN) of half the area of FK, and two half annuli, Left Half (LH) and Right Half (RH) of half the area of FK (see Fig 4A). This design ensured that size was controlled for and that we could test for differences due to inter-hemispheric connectivity. Each of the stimuli flickered at ten different driving frequencies ranging from 8.2 Hz to 17.5 Hz and the response power at the driving frequency was recorded via EEG from the electrodes over the occipital cortex.

The largest response frequency for the FK stimulus was 8.8 Hz, 10.0 Hz for the FN stimulus, and 10.0 Hz for the mean response of the LH and the RH stimuli (denoted LH+RH). The small stimuli (FN, LH+RH) were all of equivalent size and it is interesting to note that they have the same mean response peak at 10.0 Hz, suggesting an undetectable effect of intra-cortical connections. These results indicate that the cortical networks within human V1 exhibit resonance-like properties which can be observed using the SSEP. Furthermore, the negative relationship between cortical network size and resonance is clearly visible. A two-way repeated measures analysis of variance (ANOVA) was performed using the two factors: driving frequency and stimulus type for individual response powers at the two peak frequencies 8.8 Hz and 10.0 Hz. There was no main effect of frequency or stimulus on response power, but a significant interaction was found for stimulus types FK as compared to FN (F = 5.5, p = 0.02) and FK compared to LH+RH (F = 3.82, p = 0.03). These results provide evidence that a larger cortical network size results in a lower resonance frequency. The grand average scalp topographies are given for the four conditions at a single stimulus frequency (9.3 Hz). The power at the stimulus frequency normalised by the total power at each electrode can be seen in Fig 4B.

The stimulus images were made up of green and red dots, one of which was slightly dominant; this formed the basis of an attentional task which ensured that the subjects stayed focussed and alert and in addition strengthened the signal [2,24,25]. Participants were asked to decide after each trial whether the object on the screen consisted of primarily green or red dots. The mean scores per stimulus type and frequency are shown in Fig 4D with the standard error given as bars. Individual scores ranged between 40% and 100% correct. A two-way repeated measures analysis of variance (ANOVA) was performed using the two factors: driving frequency and stimulus type for individual scores at all frequencies to test whether there was any significant effect of either of these on the subjects’ scores. We found a main effect of stimulus (F = 3.29, p = 0.02), no effect of stimulus frequency (F = 1.53, p = 0.13) and no interaction effect (F = 0.47, p = 0.99). The subjects scored highest with the largest stimulus (FK) which was expected. It can be seen that the highest number of correct scores for the FK condition was achieved for a stimulus frequency of 8.8 Hz, for the FN condition at 11.7 Hz and for the LH+RH condition 10.0 Hz. For the FK and LH+RH conditions, these exactly match the greatest response frequencies suggesting that there is a potential improvement in cognitive abilities for these cases.

## Discussion

Detailed biophysical studies have shown that even the most basic units of the neural system, neurons, possess resonance characteristics. This is due to the low-pass filtering effects of the leaky conductance channels of the cell membrane coupled with the high-pass filtering effects of the voltage-gated current systems [4]. This study was concerned with understanding whether cortical resonance is affected by global properties of the activated network. We examined the possibility that the resonance frequency of a neural network (both computational and actual) is influenced by its size. The results from our modelling work indicate that when a region of simulated cortical sheet is activated, the resonance frequency of that region is inversely proportional to its size. We found a specific relationship between resonance and the number of excitatory and inhibitory connections and the average transmission time between units within the network. The prediction of our model was confirmed experimentally in the context of the primary visual cortex using a visual steady-state evoked potential paradigm. These results suggest that resonance in the cortex is not simply the consequence of oscillatory properties of the individual neurons but that network structure plays an important role.

The modelling work indicated that the number of excitatory and inhibitory connections had the strongest effect on the resonance of the network. This is in line with recent work exploring the link between intrinsic oscillator frequency, phase-synchronisation and time-delayed coupling in a sparsely connected network of phase-oscillators [15]. Nordenfelt *et al* investigated the effect of increasing time-delays between units in the network and found that this had a negative effect on the mean oscillatory frequency output of the model. They also classified the average frequency of units within the system according to their node degree and found that as the number of connections increased, the average frequency of that unit decreased. These are in direct agreement with our results. While the model described in the aforementioned paper is a phase-oscillator model, the WC model that we used in our study provides a more realistic description of neural tissue dynamics.

The human visual SSEP experiment confirmed the model prediction and provided further evidence that network size is related to cortical resonance. Interestingly, there was no significant difference in maximum response frequency between the three smaller stimuli that we used; FN was designed to activate the same total area within V1 as each of LH and RH but it activated cortical sites within each hemisphere. We expected that the FN network would encompass longer average transmission delays than LH or RH and that this would be reflected in the resonance frequency of the network. It could be that this was the case but our frequency resolution was not fine enough to elucidate this difference due to technical limitations.

In our work we define local dynamics as describing the behaviour of a single macrocolumn within our model or within the brain. The changes we observed are due to the increased number of connections and transmission delays between local units as the size of the network grew. Although we use the term ‘global’ to describe these changes at the network level, we confine this definition spatially within the brain to the visual cortex. We assume that our SSEP signal is mainly generated by locally entrained oscillators within the visual cortex and this assumption is strengthened by the fact that we observed no difference in resonance frequency for the networks activated by (LH+RH) and FN. These were the same total area but the latter encompassed cross-hemispheric connections. There is evidence that SSEP brain resonances can be local and global and that they are generated by distributed sources throughout the brain [26]. For example, the visual SSEP is observed at frontal locations under specific experimental conditions [27–29]. It is likely that we are measuring a combination of these phenomena although it should be noted that sources contributing to our signal from regions external to the visual cortex due to higher-level processing are controlled for across stimulus types.

A final point to consider is that we included stimulation frequencies outside of the natural alpha range. The strong endogenous parietooccipital alpha rhythms that exist in the absence of any stimulation leave open the question of whether stimulating with frequencies within and outside of this range may have very different biophysical bases and could be a confounding variable in the data [30,31]. Global activity of this kind is thought to modulate local activity through mechanisms such as phase-coupling [32] which has implications for the observed SSEP signal. Given that we are not trying to measure or affect behavioural or perceptual changes we feel confident that our use of the SSEP as a frequency-tag is justified in this instance.

Other authors have found evidence of a link between the frequency characteristics of neural oscillations and the spatial extent of the cortical tissue over which they traverse. For example, a recent computational paper determined that the ‘spatial reach’ of low-frequency components of the local field potential (LFP) was much larger than that of the high-frequency components [33]. In vivo and in vitro experimental work in animals has shown that slower oscillations such as alpha generally engage larger networks over more distant regions whereas higher frequency oscillations, such as gamma, are confined to local networks [34,35]. Similar results have been found in human EEG studies; local visual processing was found to occur as fast gamma oscillations whereas binding activities involving disparate areas occurred in the slower frequency ranges [36]. In general, there seems to be a consensus that long-range communication is facilitated by low-frequency oscillations whereas local communication and synchrony is brought about by high frequency activity [37]. Our study is the first to demonstrate that the activation of networks of different size within the same cortical area results in a change in resonance frequency for those networks. Based on these findings, we suggest that resonance is in fact not simply a property of the neurons within a network but also an emergent feature strongly modulated by the network itself.

The observation of an inverse relationship between network size and frequency can have a diagnostic or predictive utility. The reorganisation of brain networks, either due to slow changes in the relative contribution/wiring of brain areas (plasticity) or due to fast modulation of their causal interactions (effective connectivity), underpin the early stages of a number of developmental (e.g. Dyslexia) and psychiatric (e.g. depression, psychosis), but most prominently neurodegenerative (e.g. Alzheimer’s or Parkinson’s) conditions, ahead of any observed changes in behaviour [38,39]. A shift in the peak steady-state response frequency would be a marker of change in connectivity. A shift to a higher frequency predicts a shrinking network, while a shift to a lower frequency suggests a denser network.

The results leave open the question of multi-sensory integration. The sensory cortices might be optimally tuned to collect information from their designated environment, but how and at which frequency bands would multi-sensory stimuli be integrated remains unclear. Although we have assumed a homogeneous network in this paper, for multi-sensory integration, a neural field model would be more suitable in order to account for the heterogeneity of the systems involved. It should also be pointed out that the original WC model has been shown under certain parameter values to generate negative fractional firing rates, hence can be physiologically unrealistic. Recent work has been done in this field to make this model more biologically plausible by explicitly incorporating background variables into the equations [32].

In conclusion, we have proposed a method for exploring resonance in cortical circuits using repetitive sensory stimuli. It is known that changes associated with several brain diseases, brain traumas, as well as normal development affect brain structure. We suggest that this tool could potentially be implemented to better understand these changes quantitatively. Future work in this area will incorporate stimulus systems capable of presenting stimuli at a finer temporal scale than used here in order to overcome the technical limitations mentioned above.

## Materials and Methods

### The Wilson-Cowan Network Model

**Fig 5:**
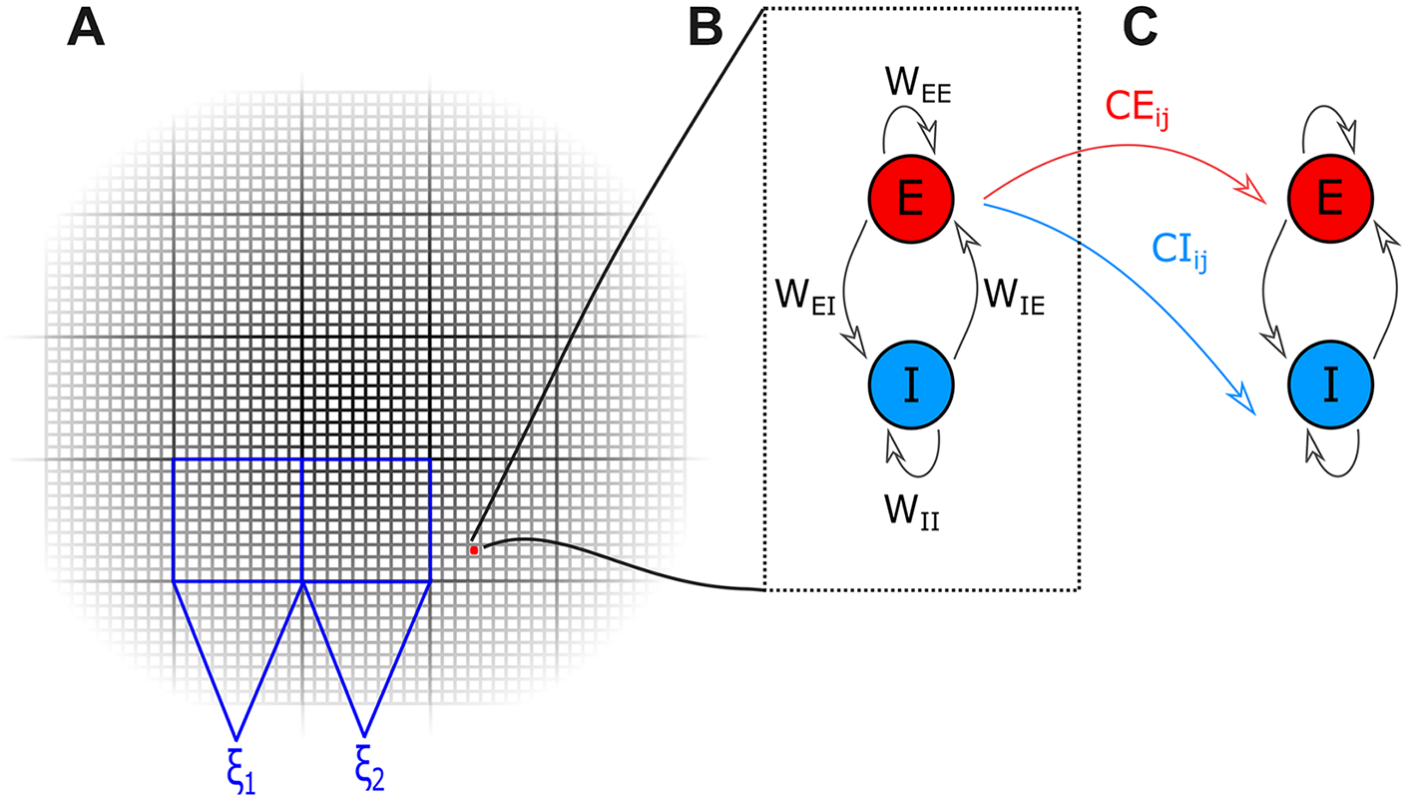
Wilson-Cowan model schematic diagram. **A** The Wilson-Cowan network model consisted of WC units fixed onto a lattice-shaped two-dimensional grid with periodic boundary conditions to simulate a homogenous patch of cortical sheet. WC units were grouped into 10x10 ‘macrocolumns’. All units within a macrocolumn received the same white noise input which was independent from the noise applied to other macrocolumns (in the diagram this is represented by the inputs ξ_1_ and ξ_2_). **B** Each WC unit contains two neural populations: excitatory (E) and inhibitory (I). Connectivity between the two populations is described by the parameters *W*_*EE*_, *W*_*II*_, *W*_*IE*_, and *W*_*EI*_, indicating the strength of connections within and between the excitatory and inhibitory populations within the unit. **C** Inter-unit connectivity is shown as red (blue) for the excitatory (inhibitory) connections. The strength of these connections are governed by the connectivity parameters contained in the independently generated connectivity matrices CE and CI, respectively. Connections are not necessarily reciprocal.

We used the WC model as the basic unit of our network [16,17]. The WC equations describe a simple model that essentially produces two types of output: oscillations and evoked responses. Network architecture was based on the model described in [18], modified with the inclusion of transmission delays and lateral inhibition of non-stimulated units. Structurally, the cortex consists of minicolumns that are approximately 50*μm*^2^ in size, which can be identified morphologically and physiologically [40]; in our model a single unit was intended to represent a minicolumn of the same size. Units were fixed onto a lattice-shaped two-dimensional grid with periodic boundary conditions to form a simulated patch of cortical sheet (see Fig 5 for a schematic diagram of the model). Each WC unit accounts for two coupled neural populations; an excitatory (E) and an inhibitory (I). The output of a WC unit is given as the action potential density of the excitatory population. The mathematical details of a single unit within the model are given in Eqs 1–3 and the parameter values for each simulation in Table 1. In the equations, the subindex *i* refers to a reference unit while the subindex *j* runs over the rest of the units in the network, and parameters without subindices have the same value for all units.

$$\tau_{E}\frac{dE_{i}}{dt}=-E_{i}+\sigma \left\{W_{EE}E_{i}+W_{IE}I_{i}+E_{0}+\sum_{j\neq i}CE_{ij}E_{j}+\;f\left(t\right)+z\;\xi \left(t\right)\right\}$$ (1)

$$\tau_{I}\frac{dI_{i}}{dt}=-I_{i}+\sigma \left\{W_{EI}E_{i}+W_{II}I_{i}+I_{0}+\sum_{j\neq i}CI_{ij}E_{j}\right\}$$ (2)

$$\sigma \left(x\right)=\frac{1}{1+e^{-m\left(x-n\right)}}$$ (3)

The strength of connections within and between the excitatory and inhibitory cell populations within a unit is described by the parameters *W*_*EE*_, *W*_*II*_, *W*_*IE*_, and *W*_*EI*_. Parameter values were fixed as *W*_*EE*_ = 23,*W*_*II*_ = 0,*W*_*EI*_ = 35,*W*_*IE*_ = −15 for all simulations as these have been shown to generate stable oscillatory behaviour when coupled with the background activity levels given below [41]. Inter-unit connectivity structure and strength are contained in the matrices *CE* and *CI*. Unless otherwise stated, the default connectivity parameters are defined as follows: when an excitatory projection exists between units *i* and *j*, *CE*_*ij*_ = 0.15 and zero otherwise; similarly, when an inhibitory projection exists between units *i* and *j CI*_*ij*_ = 0.10, and zero otherwise. In all cases, the matrices *CE* and *CI* are independently generated and connectivity is not necessarily reciprocal. The local excitatory and inhibitory connection strengths were set equal to those used in [18] which in turn were based on estimations from the biology in [42]. The effect of changing these variables on model output can be seen in S2 Fig.

The background activity levels are given by *E*_0_ and *I*_0_ for the excitatory and inhibitory populations respectively. These parameter values were fixed as *E*_0_ = 0.5 and *I*_0_ = −5 for all simulations.

The response function, *σ*, for each population is defined by a sigmoidal function, given in Eq 3, which is increasing in the interval *x*∈(−∞,∞); *m* controls the steepness of the curve and *n* the offset from 0. Parameter values were fixed at *m* = 1 and *n* = 4 for all simulations. These specific values were chosen to generate oscillatory output as in [41].

The WC unit has an intrinsic resonance frequency that can be controlled via the excitatory and inhibitory time constants(*τ*_*E*_ and *τ*_*I*_) [43,44]. The spectral response of one unit as a function of the time constants is reported in the supplementary material. The time-constants for each unit were fixed for all experiments as *τ*_*E*_ = 0.014,*τ*_*I*_ = 0.013 *ms*.

The extrinsic noise applied to the excitatory population of each unit is denoted by *ξ*(*t*), the variance of the noise was controlled by a multiplicative factor, *z*, and the repetitive stimulus *f*(*t*) are all described in detail in the next sections.

### Inter-unit Connectivity

Within the network there are excitatory and inhibitory local connections. The strengths of these connections are contained in the connectivity matrices *CE* and *CI*. Excitatory connections connect the excitatory population of one unit to the excitatory population of another, whereas inhibitory connections connect the excitatory population of one unit to the inhibitory population of another. The probability that a unit is forward-connected to any other was determined via a Gaussian fall-off that decreased with distance and calculated via a multivariate random number generator which selected numbers from a normally distributed set with mean *μ* = 0 (centred on each unit) and standard deviation *ϑ* = 250 μm with a maximum cut-off of 700 μm as in [18]. This was based on the fact that most local connections are found within 500 μm of the cell body (see [42] for a review of the anatomical literature). In addition, random excitatory long-range connections were generated from a uniform distribution with probability fixed such that the long range connections constituted approximately 25% of overall connections as described in [42]. The transmission delay between units was calculated as being proportional to the Euclidean distance between them on the grid.

Activity was confined to the stimulated area by simulating lateral inhibition. This was achieved by setting all excitatory connections from the activated area to the surrounding area to 0 (*CE*_*ij*_ = 0) and all inhibitory connections from the activated area to the surrounding area to 1 (*CI*_*ij*_ = 1). This was based on the experimental work described in [21,22].

### Model Input

#### Randomly generated white noise input

We assumed the architectural organisation detailed in [18] regarding noise input; units were grouped into 10x10 ‘macrocolumns’ receiving the same noise input from external sources. The extrinsic noise input, denoted by ***ξ***(***t***), was sampled from Gaussian distributed white-noise with zero mean and unit variance. This was intended to mimic the background afferent activity from other regions. The variance of the noise was controlled by a multiplicative factor, ***z***. See S2 Fig for a description of how ***z*** affects the model output. Values for ***z*** are given in Table 1 for each simulation. The Euler-Murayama method for the integration of stochastic differential equations was used with an integration step of 1 ms; we checked that no change in results was obtained by either doubling or halving the integration step.

#### Repetitive input

In the case of the stimulus-driven network, a repetitive spiking input was added to the randomly generated white noise using Eq 4 [45]. The parameter values for ***f***(***t***) were set as follows: ***q* = 0.5, *n* = 3.0**, and ***w* = 3.7**.

$$f\left(t\right)=q{\left(\frac{t}{w}\right)}^{n}e^{\frac{-t}{w}}$$ (4)

### Simulations

#### Simulations—Resonance characteristics of sub-networks within the model

We generated a network of 50x50 identical units connected as described above, and then stimulated sub-networks within this larger network in order to match our computational work as closely as possible to the intended experimental work. We applied an external driving frequency that was systematically varied between 10.0 Hz and 15.0 Hz in steps of 0.5 Hz and applied this input to units belonging to either a centrally located 10x10 unit sub-network, or a 30x30 unit sub-network. We generated 100 trials of 2000 time samples each for each condition. Mean response frequency and standard deviation were calculated using the bootstrapping method [46]. For each trial, a power series of the averaged time-series across all activated units within the network was calculated from the squared complex conjugate of the Fourier coefficients, and normalised by the total area under the curve. For each condition, N = 100 power series were sampled with replacement 1000 times from the data. An average power spectrum was calculated for each of these 1000 selections. Response power at the driving frequency, normalised to the area under the spectrum in each case, was recorded for each of the 1000 averaged spectra. These values were used to calculate mean and standard deviation for each condition.

We use the term ‘delay’ in this manuscript to describe the length of time that it takes a signal to propagate along the axon from one unit to another. In the brain, this delay depends on the distance between the units and the conduction velocity of the connecting axons. Conduction velocity in turn depends on myelination, the width of the axons and the positioning of the nodes of Ranvier [47]. Physiologically, transmission delay is the result of a delicate interplay between these factors with some authors suggesting that conduction velocity is actually plastic allowing the brain to finely tune signal arrival times between specific neuronal areas [27]. For the purposes of our study, we assume that the conduction velocity is fixed and therefore the delay is proportional to the distance between two connected units on a Euclidean plane. We base the above assumption on the fact that in the primary visual cortex we are working with a homogenous network essentially focussing on lateral connections across the cortical surface.

Under constant conduction velocity, the transmission delay between minicolumns is representative of distance. We used a standard delay of 10 ms per minicolumn as has been used in other modelling studies, namely [44,48] which in the latter case was based on the anatomical data derived from macaque monkeys in [49]. We acknowledge that conduction velocities are rarely fixed in the brain and depend on a number of factors. Distributed delays are known to modulate the EEG power spectrum although it has been shown that a discrete delay is a good first approximation [50]. Therefore, here as in other papers we assume fixed conduction velocity in order to simplify the simulations and our understanding of the model behaviour. Investigations into the effect of distributed delays on network behaviour remain an open area of research.

#### Simulations—Impact of network properties on the resonance frequency

We examined the connectivity profiles of successively larger activated sub-networks within a fixed global framework consisting of 100x100 units with connectivity architecture as described previously. The smallest sub-network was a 2x2 centrally located network followed by a 4x4 centrally located sub-network and so on until the entire network 100x100 units was included. We computed the average node degree per sub-network by counting the number of connections (both excitatory and inhibitory) between all units that fell within the bounds of the specified sub-network, as well as the average transmission delay between those connected nodes with all other parameter values fixed (see Table 1).

#### Simulations—Fixed node degree

Next, we conducted a numerical analysis on the effect of increasing delay on the resonance of a 30x30 unit network. We varied the transmission delay between two adjacent units on the lattice from 10 to 100 in steps of 10 (all other parameters remained fixed) while stimulating the network with a repetitive input varied between 12 Hz and 14 Hz in steps of 0.2 Hz. We generated 100 trials of 5000 time samples each for each condition. Time-series were averaged across the network and a power series was calculated for each trial. In order to calculate values for the mean and standard deviation, we employed the bootstrapping method described previously. N = 100 power series were sampled with replacement for each condition and each frequency 1000 times. The driving frequency with maximal response for each of the 1000 sampled-sets was recorded and this data was used to calculate the mean and standard deviation per condition.

#### Simulations—Fixed delay

In order to investigate the effect of increasing local node degree on the model output we assumed instantaneous transmission between units so that the average transmission delay did not increase with the extra connections. However, within the network there are both excitatory and inhibitory local connections between units. Therefore, we conducted a simulation experiment where the excitatory (inhibitory) local connections per unit were fixed to a value of 5 while the inhibitory (excitatory) local connections were increased from 0 to 9 while stimulating the network with a repetitive input varied between 12 Hz and 17 Hz in steps of 0.2 Hz. We generated 1000 trials of 5000 time samples each for each condition. The frequency of maximal response for each of the 1000 sampled-sets was recorded, as above, and this data was used to calculate the mean and standard deviation per condition. The standard deviation of the noise was increased to ***z* = 0.07** to prevent damping at higher levels of connection density.

The mean node degree is defined as the average number of connections per unit. In the following simulations, we define excitatory (inhibitory) node degree to describe the average number of forward local excitatory (inhibitory) connections for each unit.

### Model Choice

We considered two model-types before embarking on this work: the neural field model and the network model. Field models treat the neural tissue as continuous in both space and time [51]. Spatial attributes such as size are intrinsic to the model and can therefore be adjusted easily allowing in-depth investigation into the effects on model behaviour. Conversely, a network model describes the neural tissue as an interconnected network of nodes. The latter method is often used to examine the specific effect of different network structures and due to its relative computational efficiency can be used to model local and global, including whole-brain, dynamics (see [52] for example). Connectivity topology within the brain consists of a mix of dense nearest-neighbour connections and sparse long-distance projections [53]. In light of this, it has been suggested that cortical models which combine these two methodologies may be the most realistic. That is, local short-range connections are thought to be extremely dense such that local properties of the brain tissue are well-described by the continuum case while long-range connections are known to be sparse and specific and therefore easier to describe using a discrete approach. There has been recent work in developing neural field models that take into account heterogeneous lateral connections [53,54]. We base our model on that proposed by [42] and implemented by [18]; a network model consisting of dense local connections which approach the continuous case with the inclusion of a percentage of sparse long-range connections between distant units. It should be noted that neural field models are a generalisation of the neural mass models which assume a continuous tissue structure rather than discretely distributed interconnected neural masses, see [55] for details. However, field models are much more computationally expensive and in the context of our study we did not feel that approach was justified.

### Experimental Work

#### Ethics statement

Fourteen healthy participants (two men; 22–32 years) with normal or corrected-to-normal vision gave informed consent to participate in this experiment. Data from one participant was excluded because of excessive artefacts in the data. This study was approved by the University of Manchester Ethics Committee.

#### Stimuli

The visual stimuli were designed and presented using a visual stimulation generator (ViSaGe, Cambridge Research Systems Co. Ltd.) which ensured precise and accurate timings of the flashing images. In total, four image types were presented, a Full Thick annulus (FK), a Full Thin annulus (FN) which forms exactly half the area of FK, and two half annuli, Left Half (LH) and Right Half (RH) which again form exactly half the area of FK. The annuli were made up of green and red dots, one of which was slightly dominant and participants were asked at the end of each trial to report which.

The precise flicker frequencies were determined by the screen refresh rate of the presentation system which was 140 frames per second resulting in a frame time of 7.143 ms. Using cycle lengths of integer values between 8 and 17 frames resulted in ten stimulation frequencies of 8.24 Hz, 8.75 Hz, 9.33 Hz, 10.00 Hz, 10.77 Hz, 11.67 Hz, 12.73 Hz, 14.00 Hz, 15.56 Hz, 17.50 Hz. To generate the flicker, the image screen was presented for the first five frames of the cycle, with the remaining frames showing only the background screen colour such that the total number of frames summed to the desired cycle length.

Participants were asked to fixate on a small marker at the centre of the screen for the duration of the trial and try to detect whether the flashing image in their peripheral view was primarily made of green or red dots. The attentional task was to ensure that the participants did not get bored and also the SSEP signal has been shown to increase with attention [1,2,24,25,56–59]. Preceding each trial, a screen with the word ‘Blink’ was presented for 1s. The purpose of this was to remind the participant to blink before the trial began and refrain from doing so during the stimulus presentation. Each trial lasted 3 seconds and was repeated 10 times per frequency (presented randomly). After each trial, the participant was asked to make a choice by pressing a keyboard key as to the dominant colour of the image. A break of 3 seconds was included between trials. At the end of a block, which consisted of all the trials for a specific image type, there was a longer break with water offered to the participant before the next image type was loaded. Data were recorded in four blocks lasting ∼10 min each with a total of 100 trials per image type (10 for each frequency). After all four blocks were complete, we recorded two minutes of resting state with eyes open and two minutes of resting state with eyes closed.

#### EEG recording

The experiment took place in a dark, sound-proof, electrically-shielded room. The participants sat comfortably on a chair facing the stimulus screen and were asked to avoid any movements or eye-blinks during the trials. Frequent breaks and continual monitoring of the participants appeared to successfully ensure their on-going comfort and continued attention. The continuous EEG signals were recorded using the 64-electrode ActiveTwo system (BioSemi, Amsterdam, Netherlands) with Actiview acquisition software (BioSemi, Netherlands). Impedance of each electrode was maintained below 5 kΩ for the duration of each experiment. The signal was digitized at 512 Hz with a pass band from 0.01 to 100 Hz. Horizontal and vertical electro-oculograms were recorded using separate electrodes placed above and below the right eye and at the outer canthi of both eyes. The precise stimulus markers were recorded by the Biosemi system.

#### Preprocessing

The data were taken offline for full analysis using SPM (http://www.fil.ion.ucl.ac.uk/spm/) and EEGLAB (http://sccn.ucsd.edu/eeglab/) toolboxes implemented in Matlab (Mathworks, http://www.mathworks.com). The data were re-referenced to an average reference and filtered using a high-pass of 1 Hz and then a low-pass of 80 Hz. ICA was performed to remove ocular artefacts using EEGLAB [60]. The data epochs were extracted beginning 500 ms after stimulus onset and lasting for 2500 ms per trial. Vertical and horizontal eye movements were identified using a criterion of ±100 μV using the inbuilt artefact rejection algorithms and following that, every trial was inspected by eye to ensure data quality. Next, the data from only the relevant electrodes was averaged per trial, these included O1, O2, Oz and POz, determined from an analysis over all participants to find the channels exhibiting the average greatest response power across all stimuli.

#### Data analysis

For each condition, the power spectrum was calculated from the squared complex conjugate of the Fourier coefficients and normalised by the total area under the curve using 2.5 seconds of averaged EEG data. The SSEP power for the trial was calculated as the maximum squared amplitude of the Fourier coefficients for the three frequency values closest to the stimulus frequency and these were normalised by total power under the curve for between-participant comparison.

## Supporting Information

S1 Fig Resonance properties and entrainment of a single-unit. **A** The effect of varying the excitatory and inhibitory time constants (**τ**_**e**_ and **τ**_**i**_) on the spectral output of a single unit responding to a white-noise input is given, as previously shown in [43,44]. Left figure shows the mean peak frequency of a single unit and the power of the mean peak frequency on the right. The time-constants were varied between 0.01 and 0.02. It is the ratio of these two parameters that drives the intrinsic resonance of the unit and a clear negative correlation exists between amplitude and frequency as expected. **B** By varying the excitatory and inhibitory time constants of the model as indicated, the intrinsic resonance of the model can be tuned to a specific frequency. The blue curve in each case shows the averaged power spectra for generated spontaneous behaviour. A clear shift in resonance (from left to right 7.8 Hz, 9.0 Hz, 10.2 Hz, 11.2 Hz, 12.6 Hz) is observed as **τ**_**i**_ is decreased from 0.017 to 0.013 with **τ**_**e**_ fixed at 0.018. The dashed red curve gives the mean response power to an external driving frequency that was systematically varied between 6 Hz and 13 Hz in steps of 0.2 Hz for each case. It can clearly be seen that the entraining frequency exhibits maximum response power at the resonant frequency for each case, as expected.
(DOCX)

S2 Fig Effect of extrinsic noise and inter-unit connectivity strengths on the model output. **A** The mean peak frequency and the power of the mean peak frequency of the 30x30 unit network as a function of increasing the standard deviation of the random white noise input **(*z***) was calculated using the bootstrap method previously described with 100 trials. The mean peak frequency increases linearly with noise levels **(*R***^**2**^ = **0.95, *p* < 0.0001)**(decreasing for values greater than shown here) and a stochastic resonance effect can be seen for ***z*** = **0.05(*R***^**2**^ = **0.49, *p*** = **0.09).** The peak of this curve shifts depending on the various network factors such as network size, mean transmission delay, mean node degree etc. **B** Mean peak frequency for a network as described above as a function of increasing the two inter-unit connectivity parameters (***cs***_***e***_ and ***cs***_***i***_) for the excitatory and inhibitory connectivity strengths (with ***z*** = **0.05** fixed) calculated using the bootstrap method previously described with 500 trials. Increasing the strength of the excitatory or the inhibitory connections increased the peak frequency of the network measured as a response to white-noise input. The power of the peak frequency for values of either of the connectivity constants greater than **0.3** amplitude is increasingly damped.
(DOCX)
