# Supplementary material for: Cortical Resonance Frequencies Emerge from Network Size and Connectivity
Source: PLoS Comput Biol. 2016 Feb 25;12(2):e1004740. doi: 10.1371/journal.pcbi.1004740 (PMC4767278; doi:10.1371/journal.pcbi.1004740)
Supplement: S1 Fig — A The effect of varying the excitatory and inhibitory time constants (τe and τi) on the spectral output of a single unit responding to a white-noise input is given, as previously shown in [43,44]. Left figure shows the mean peak frequency of a single unit and the power of the mean peak frequency on the right. The time-constants were varied between 0.01 and 0.02. It is the ratio of these two parameters that drives the intrinsic resonance of the unit and a clear negative correlation exists between amplitude and frequency as expected. B By varying the excitatory and inhibitory time constants of the model as indicated, the intrinsic resonance of the model can be tuned to a specific frequency. The blue curve in each case shows the averaged power spectra for generated spontaneous behaviour. A clear shift in resonance (from left to right 7.8 Hz, 9.0 Hz, 10.2 Hz, 11.2 Hz, 12.6 Hz) is observed as τi is decreased from 0.017 to 0.013 with τe fixed at 0.018. The dashed red curve gives the mean response power to an external driving frequency that was systematically varied between 6 Hz and 13 Hz in steps of 0.2 Hz for each case. It can clearly be seen that the entraining frequency exhibits maximum response power at the resonant frequency for each case, as expected. (DOCX) [file pcbi.1004740.s001.docx]

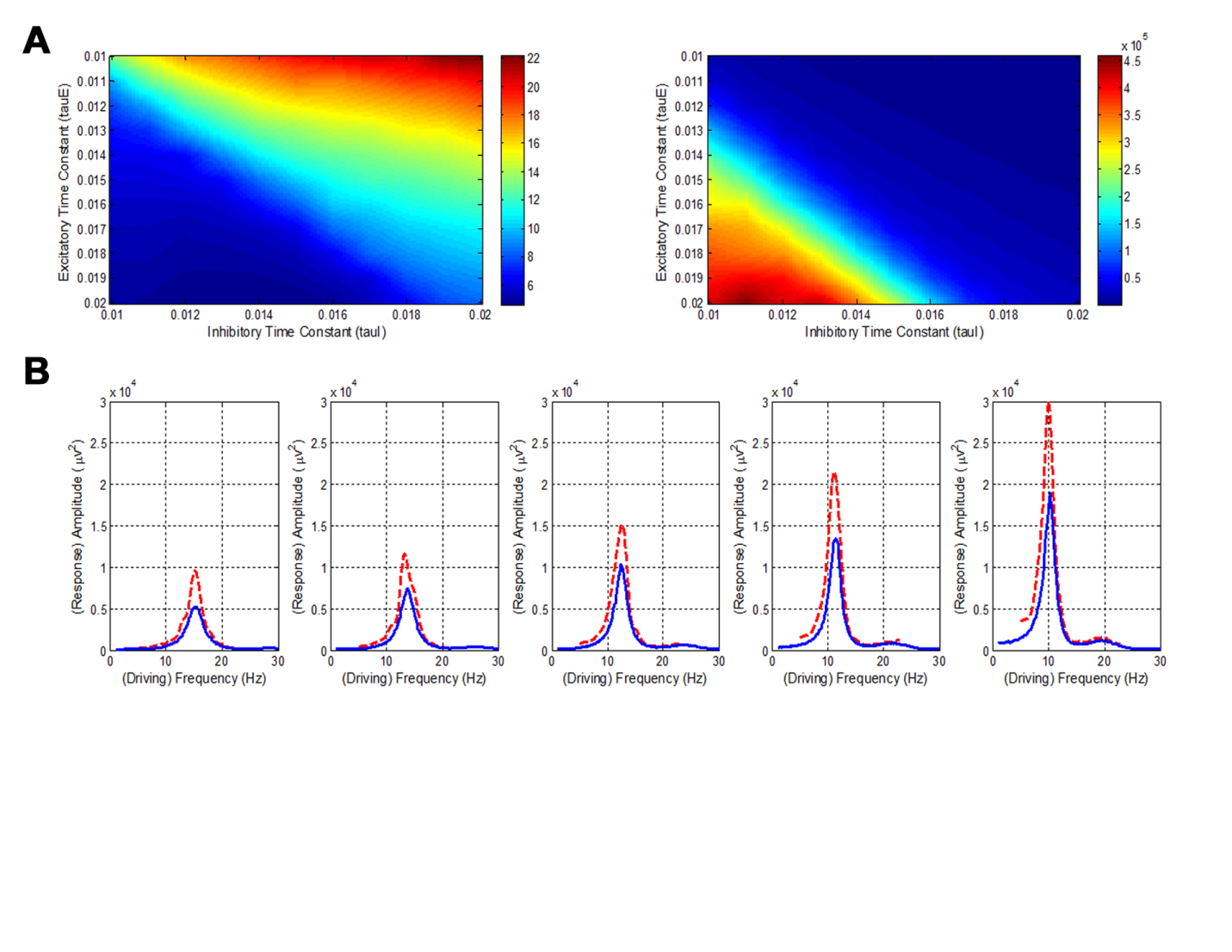


**S1. Resonance Properties and Entrainment of a single-unit**

**A** The effect of varying the excitatory and inhibitory time constants ($\tau_{e}$ and $\tau_{i}$) on the spectral output of a single unit responding to a white-noise input is given, as previously shown in [[1](#_ENREF_1),[2](#_ENREF_2)]. Left figure shows the mean peak frequency of a single unit and the power of the mean peak frequency on the right. The time-constants were varied between 0.01 and 0.02. It is the ratio of these two parameters that drives the intrinsic resonance of the unit and a clear negative correlation exists between amplitude and frequency as expected. **B** By varying the excitatory and inhibitory time constants of the model as indicated, the intrinsic resonance of the model can be tuned to a specific frequency. The blue curve in each case shows the averaged power spectra for generated spontaneous behaviour. A clear shift in resonance (from left to right 7.8 Hz, 9.0 Hz, 10.2 Hz, 11.2 Hz, 12.6 Hz) is observed as $\tau_{i}$ is decreased from 0.017 to 0.013 with $\tau_{e}$ fixed at 0.018. The dashed red curve gives the mean response power to an external driving frequency that was systematically varied between 6 Hz and 13 Hz in steps of 0.2 Hz for each case. It can clearly be seen that the entraining frequency exhibits maximum response power at the resonant frequency for each case, as expected.

1. Wang Y (2013) Multi-scale modelling of epileptic seizure rhythms as spatio-temporal patterns: University of Manchester.

2. David O, Friston KJ (2003) A neural mass model for MEG/EEG: coupling and neuronal dynamics. Neuroimage 20: 1743-1755.
