# Supplementary material for: Cortical Resonance Frequencies Emerge from Network Size and Connectivity
Source: PLoS Comput Biol. 2016 Feb 25;12(2):e1004740. doi: 10.1371/journal.pcbi.1004740 (PMC4767278; doi:10.1371/journal.pcbi.1004740)
Supplement: S2 Fig — A The mean peak frequency and the power of the mean peak frequency of the 30x30 unit network as a function of increasing the standard deviation of the random white noise input (z) was calculated using the bootstrap method previously described with 100 trials. The mean peak frequency increases linearly with noise levels (R2 = 0.95, p < 0.0001)(decreasing for values greater than shown here) and a stochastic resonance effect can be seen for z = 0.05(R2 = 0.49, p = 0.09). The peak of this curve shifts depending on the various network factors such as network size, mean transmission delay, mean node degree etc. B Mean peak frequency for a network as described above as a function of increasing the two inter-unit connectivity parameters (cse and csi) for the excitatory and inhibitory connectivity strengths (with z = 0.05 fixed) calculated using the bootstrap method previously described with 500 trials. Increasing the strength of the excitatory or the inhibitory connections increased the peak frequency of the network measured as a response to white-noise input. The power of the peak frequency for values of either of the connectivity constants greater than 0.3 amplitude is increasingly damped. (DOCX) [file pcbi.1004740.s002.docx]

**
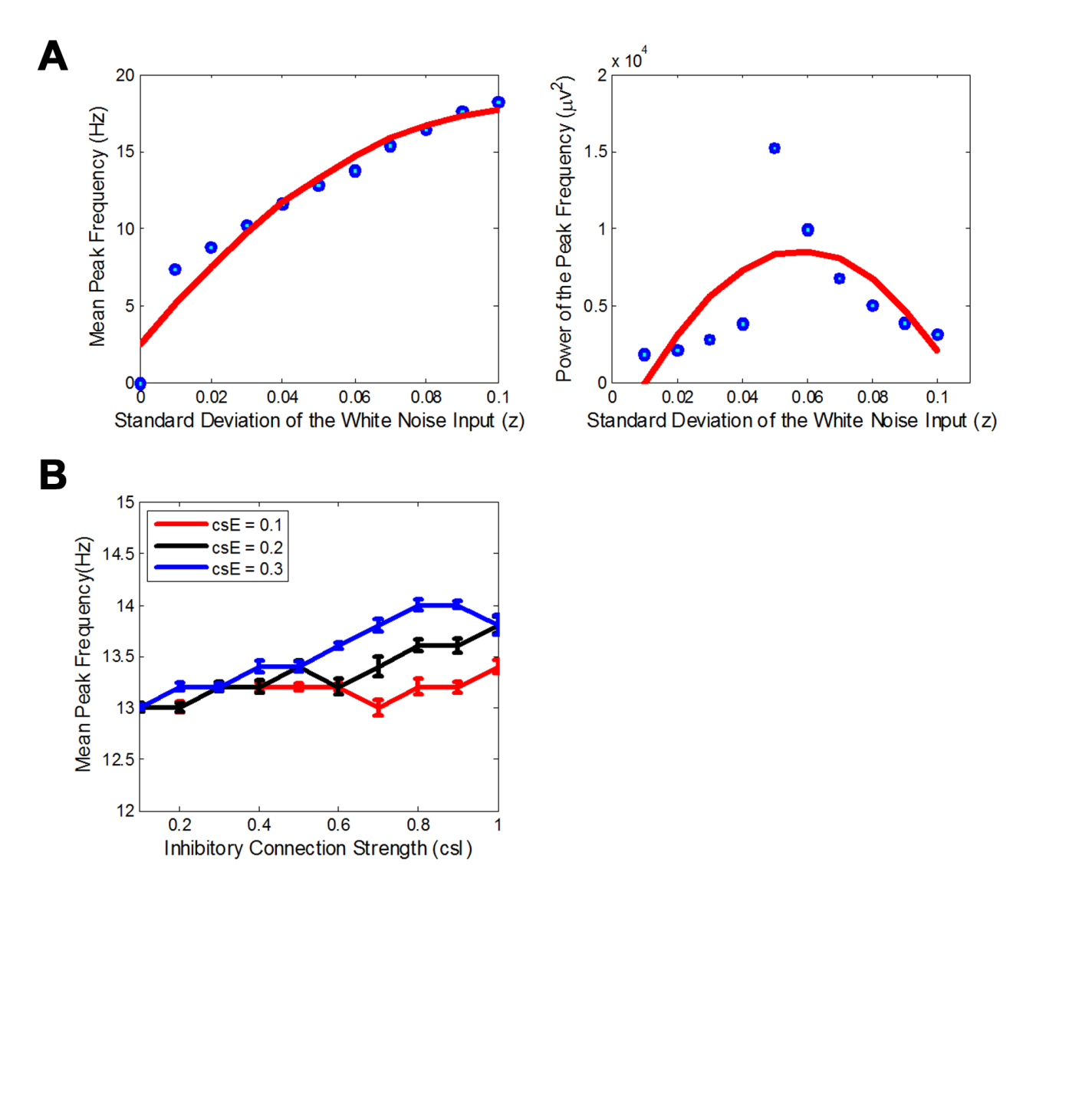
**

**S2. Effect of extrinsic noise and inter-unit connectivity strengths on the model output**

**A** The mean peak frequency and the power of the mean peak frequency of the 30x30 unit network as a function of increasing the standard deviation of the random white noise input $(z$) was calculated using the bootstrap method previously described with 100 trials. The mean peak frequency increases linearly with noise levels ($R^{2}=0.95,p<0.0001$) (decreasing for values greater than shown here) and a stochastic resonance effect can be seen for $z=0.05 (R^{2}=0.49,p=0.09).$ The peak of this curve shifts depending on the various network factors such as network size, mean transmission delay, mean node degree etc. **B** Mean peak frequency for a network as described above as a function of increasing the two inter-unit connectivity parameters (${cs}_{e}$ and ${cs}_{i}$) for the excitatory and inhibitory connectivity strengths (with $z=0.05$ fixed) calculated using the bootstrap method previously described with 500 trials. Increasing the strength of the excitatory or the inhibitory connections increased the peak frequency of the network measured as a response to white-noise input. The power of the peak frequency for values of either of the connectivity constants greater than $0.3$ amplitude is increasingly damped.
